# Effect of Post-Welding Aging Treatment on the Microstructure and High-Temperature Properties of Inertia Friction Welded GH4065A Joint

**DOI:** 10.3390/ma16103639

**Published:** 2023-05-10

**Authors:** Sheng Cao, Xiaoguang Li, Jiatao Liu, Chunbo Zhang, Jun Zhou, Lei Cui

**Affiliations:** 1 School of Materials Science and Engineering, Tianjin University, Tianjin 300350, China; 2 Tianjin Key Laboratory of Advanced Joining Technology, Tianjin 300350, China; 3 AECC Shenyang Liming Aero-Engine Co., Ltd., Shenyang 110043, China; 4 Harbin Welding Institute Co., Ltd., Harbin 150028, China

**Keywords:** GH4065A alloy, inertia friction welding, aging treatment, microstructure, high-temperature properties

## Abstract

In this study, post-welding aging treatments were applied to a novel Ni-based superalloy GH4065A inertia friction welding (IFW) joint to improve its high-temperature properties. The effect of aging treatment on the microstructure and creep resistance of the IFW joint was systematically investigated. The results indicated that the original $\gamma^{\prime }$ precipitates in the weld zone almost completely dissolved during the welding process, and fine tertiary $\gamma^{\prime }$ precipitated during the subsequent cooling process. Aging treatment did not significantly change the characteristics of grain structures and primary $\gamma^{\prime }$ in the IFW joint. After aging, the size of tertiary $\gamma^{\prime }$ in the weld zone and secondary $\gamma^{\prime }$ in the base material increased, but their morphology and volume fraction did not change evidently. After 760 °C, 5 h aging treatment, the tertiary $\gamma^{\prime }$ in the weld zone of the joint grew from 12.4 nm to 17.6 nm. Correspondingly, the creep rupture time of the joint at 650 °C and 950 MPa increased from 7.51 h to 147.28 h, which is about 19.61 times higher than that of the as-welded joint. The creep rupture was more likely to occur in the base material instead of the weld zone for the IFW joint. This revealed that the creep resistance of the weld zone was significantly improved after aging due to the growth of $\mathrm{tertiary}\;\gamma^{\prime }$. However, increasing the aging temperature or extending the aging time promoted the growth of secondary $\gamma^{\prime }$ in the base material, and meanwhile, M_23_C_6_ carbides tended to continuously precipitate at the grain boundaries of the base material. It might decrease the creep resistance of the base material.

## 1. Introduction

The turbine disk, a key hot section component in aero engines, serves in high-temperature and harsh high-pressure environments, which requires materials with excellent high-temperature properties [1,2]. Ni-based superalloys have become the preferred materials for turbine disks due to superior creep resistance, fatigue property and corrosion resistance at high temperatures [3]. $\gamma^{\prime }$-Ni_3_(Al, Ti) precipitate is the main strengthening phase of Ni-based superalloys, and its size, shape and volume fraction can obviously affect the high-temperature properties of the alloy [4,5]. According to the formation condition and dimension characteristic, $\gamma^{\prime }$ is generally classified into primary $\gamma^{\prime }$, secondary $\gamma^{\prime }$ and tertiary $\gamma^{\prime }$ [6,7,8,9]. The primary $\gamma^{\prime }$ with a size of 1~10 μm inhibits grain boundary migration and grain growth during the forging process. The secondary $\gamma^{\prime }$ is formed during the cooling processes of forging and solution treatment. The distributed secondary $\gamma^{\prime },$ with a size of 50–500 nm, can effectively pin dislocations and contribute to a huge precipitation-strengthening effect. After welding or aging treatment, the tiny tertiary $\gamma^{\prime }$ (<50 nm) plays a certain role in precipitation strengthening.

GH4065A, named according to GB/T 14992-2005, is a novel Ni-based superalloy for the new generation turbine disk [10]. GH4065A, with a high-volume fraction of $\gamma^{\prime }$ (~42%), possesses excellent high-temperature properties and can reach a maximum service temperature of 750 °C [6,11]. Therefore, GH4065A is regarded as one of the important materials for the turbine disk of new-generation aero engines.

Currently, inertia friction welding (IFW) is the primary welding method for Ni-based superalloy turbine disks. During the welding process, severe plastic deformation of the weld material contributes to sufficient dynamic recrystallization and tiny equiaxed grains formation [12,13]. However, the original $\gamma^{\prime }$ dissolves into the matrix and only tiny $\gamma^{\prime }$ re-precipitates during the subsequent cooling process, resulting in strength descent of the welded joint. Press et al. [14] studied the precipitation evolution of the IN718, U720Li and RR1000 IFW joints and the corresponding influence on the joint strength. It was found that the $\gamma^{\prime }$/$\gamma^{\prime \prime }$ (volume fraction ~25%) in the weld zone of the IN718 IFW joint almost completely dissolved into the matrix during the welding process. However, they barely re-precipitated during the cooling process. An obvious reduction in the microhardness of the weld zone was observed. For the U720Li and RR1000 (~40–50% $\gamma^{\prime }$) IFW joints, the weld zone re-precipitated a large number of fine $\gamma^{\prime }$ during the cooling process. The yield strength of the U720Li and RR1000 joints decreased by 100–200 MPa.

Post-welding heat treatment (PWHT) can efficiently optimize the size, morphology, and volume fraction of the strengthening phase in alloy and is considered to be an important method to improve the mechanical properties of Ni-based superalloy IFW joint. [15,16]. Masoumi et al. [17] studied the effect of solution and aging heat treatment on the AD730^TM^ linear friction welded joint. It was found that $\gamma^{\prime }$ in the heat affected zone re-precipitated after the 1080 °C solution treatment for 4 h and 730 °C aging treatment for 8 h. At room temperature and high temperature (650 °C), the ultimate tensile strength of the PWHTed joint increased by ~130 MPa and ~200 MPa, respectively. Zhou et al. [7] conducted a 760 °C aging treatment for the FGH96 IFW joint. After aging, the tertiary $\gamma^{\prime }$ in the weld zone grew from 16 nm to 20 nm, and the yield strength of the joint at room temperature increased by ~80 MPa. These studies indicate that PWHT can improve the tensile properties of Ni-based superalloy IFW joints. However, the effect of PWHT on the creep resistance of Ni-based superalloy IFW joint has not been investigated. Therefore, for the novel Ni-based superalloy GH4065A, it is significant to further investigate the impact of PWHT on the microstructure and creep resistance of the IFW joint. It is worth noting that the purpose of PWHT is to optimize the weld zone microstructure, thus, improve the high-temperature properties of the IFW joint. In order not to change the microstructure of the IFW joint base material, the PWHT in this study is carried out near the service temperature (730/760 °C) for a short time (5/8 h).

In this study, the microstructure and creep property of defect-free GH4065A IFW joint after three different post-welding aging treatments were studied. The precipitation evaluation in typical regions of the GH4065A IFW joint was characterized by Optical microscopy (OM), scanning electron microscopy (SEM), and transmission electron microscopy (TEM). The creep resistance of the GH4065A IFW joint at high temperatures was tested. The evolution behavior of $\gamma^{\prime }$ and the corresponding strengthening mechanism were analyzed. The purpose of the present study is to provide a reference for improving the high-temperature properties of the GH4065A IFW joint.

## 2. Materials and Methods

**Figure 1:**
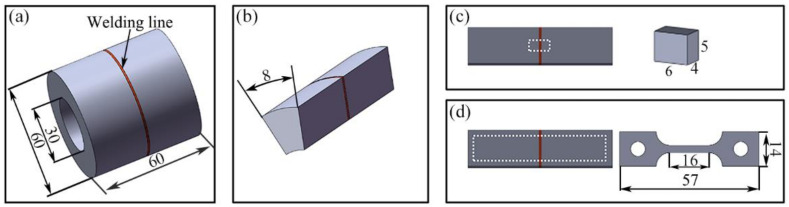
Schematics of the GH4065A IFW joint (**a**), PWHT sample (**b**), sampling location and size of the metallographic sample (**c**) and creep specimen (**d**) (unit: mm).

The chemical composition of GH4065A is listed in Table 1. Firstly, the forged GH4065A was subjected to a solution and aging treatment. Subsequently, the base material was machined into several rings with an axial length of 30 mm, an outside diameter of 60 mm, and an inside diameter of 30 mm for the IFW experiment. The constant welding parameters include rotational inertia 388 kg·m^2^, rotational speed 500 rpm, and axial pressure 350 MPa. The IFW joint is shown in Figure 1a. After welding, fan ring samples with an arc length of 8 mm (Figure 1b) were cut from the IFW joint by spark cutting. These samples were divided into three groups for aging treatment with varying temperatures and time. The first group was aged at 730 °C for 8 h; the second and third groups were aged at 760 °C for 5 h and 8 h, respectively. Each group had two PWHT samples, which were used to prepare metallographic samples and creep specimens, respectively. The aging treatment was conducted in a vacuum environment at a heating rate of 10 °C/min, followed by cooling in air.

The metallographic samples and creep specimens were cut from the as-welded and PWHTed joints by spark cutting, and their sampling location and size are shown in Figure 1c,d, respectively. The thickness of the creep specimen was 1.5 mm. For each aging condition, three creep specimens were prepared for testing. Referring to ASTM E139-2006, creep tests were carried out with DDL50 electronic creep rupture tester (Sinotest Equipment Co., Ltd., Changchun, China) at 650 °C and 950 MPa. The measurement accuracy of strain and temperature in the creep test is 0.002 mm and 0.1 °C, respectively.

The macrographs of metallographic samples and fracture locations of crept specimens were observed by Smartzoom5 OM. The OM samples were firstly mechanically ground, mechanically polished, and then etched with a mixture of 100 mL C_2_H_5_OH, 100 mL HCl, and 20 g CuCl_2_ for 30 s. The HCl used to prepare the etching solution was a saturated water solution with a concentration of 37%. The microstructure was characterized by JSM-7800 SEM (JEOL Japan Electronics Co., Ltd., Tokyo, Japan) with electron backscatter diffraction (EBSD). The SEM specimens were firstly mechanically ground, then electropolished with a solution of 10% HClO_4_ and 90% C_2_H_5_OH (by volume) at 20 V for 10 s, and finally electro-etched with a mixture of 20% H_2_SO_4_ and 80% C_2_H_5_OH (by volume) at 5 V for 5 s. The EBSD specimens were electropolished with a mixture of 20% H_2_SO_4_ and 80% CH_3_OH (by volume) at 15 V for 10 s. Tecnai G2F30 TEM (FEI Company, Hillsboro, OR, USA) was used to characterize $\gamma^{\prime }$. The TEM specimens were firstly prepared into discs with a diameter of 3 mm and a thickness of 50 μm and subsequently electropolished with a solution of 10% HClO_4_ and 90% C_2_H_5_OH (by volume) at −25 °C. Photoshop2020 software was used to summarize the geometric characteristics of $\gamma^{\prime }$ precipitate. Specifically, the size distribution and area fraction of $\gamma^{\prime }$ was calculated from five SEM or TEM images, each image was counted three times to reduce the error, and the calculation of area fraction was approximated as the volume fractions of $\gamma^{\prime }$ in this work [18].

## 3. Results

### 3.1. Microstructure of the As-Welded Joint

**Figure 2:**
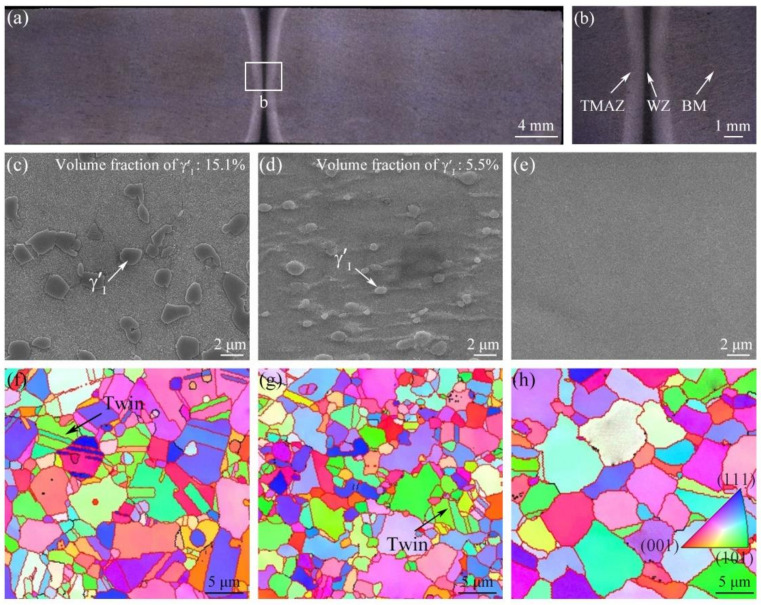
Low (**a**) and high (**b**) magnified micrographs of the GH4065A IFW joint, SEM images (**c**–**e**) and IPFs (**f**–**h**) of BM (**c**,**f**), TMAZ (**d**,**g**), and WZ (**e**,**h**) (Subfigure (**b**) is the enlarged view of area b in subfigure (**a**)).

The cross-sectional observations of the as-welded GH4065A IFW joint are shown in Figure 2. As seen in Figure 2a, the joint is defect-free and exhibits good weld formation. Figure 2b shows three typical zones, including weld zone (WZ), thermal-mechanical affected zone (TMAZ), and base material (BM), which can be identified in the joint according to the differences in their microstructural characteristics. As revealed in Figure 2a,b, the widths of TMAZ and WZ increase as getting closer to the inner and outer surfaces of the joint due to more plasticized material flowing to both sides during the IFW process.

Figure 2c–e shows the microstructure of BM, TMAZ and WZ of the as-welded joint. As shown in Figure 2c, irregular-shaped primary $\gamma^{\prime }$(${\gamma^{\prime }}_{Ⅰ}$) is distributed at the γ grain boundaries in BM with an equivalent diameter of 1–3 μm and a volume fraction of 15.1%. In Figure 2d, TMAZ consists of a γ matrix and an irregular-shaped ${\gamma^{\prime }}_{Ⅰ}$ with a lower volume fraction of 5.5% and a smaller size. This is because the welding peak temperature in TMAZ (~1100–1200 °C) slightly higher than the ${\gamma^{\prime }}_{Ⅰ}$ dissolution temperature (~1110 °C) [19,20], resulting in the partial dissolution of ${\gamma^{\prime }}_{Ⅰ}$ during the IFW process. Moreover, as shown in Figure 2e, ${\gamma^{\prime }}_{Ⅰ}$ is not observed in WZ, indicating that ${\gamma^{\prime }}_{Ⅰ}$ almost completely dissolves into the γ matrix in WZ. It is because the peak temperature in WZ reaches 1300 °C during the IFW process, which is greater than the dissolution temperature of ${\gamma^{\prime }}_{Ⅰ}$ [7].

Figure 2f–h show the inverse pole figures (IPFs) of typical regions of the as-welded joint. The black and red lines in IPFs were used to denote the low-angle grain boundaries (1–15°) (LAGBs) and high-angle grain boundaries (15–180°) (HAGBs), respectively. As shown in Figure 2f, equiaxed crystals and twins are observed in BM, and the average size of grain in BM is 3.66 μm. TMAZ also consists of equiaxed crystals and twins (Figure 2g). The mean grain size of TMAZ is 2.86 μm, which is nearly 21.8% smaller than that of BM, and the number of twins reduces. It is because that dynamic recrystallization occurs in TMAZ during the IFW process. In Figure 2h, WZ is mainly composed of recrystallized grains without twins. The average grain size of WZ is 4.88 μm, which is nearly 33.3% larger than that of BM. The formation mechanism of larger grains in WZ is that the growth of the recrystallized grains in WZ is not inhibited by ${\gamma^{\prime }}_{Ⅰ}$, which almost dissolves into the γ matrix [8]. Meanwhile, the grain boundaries in BM, TMAZ and WZ of the as-welded joint are primarily HAGBs, and the HAGBs proportions in BM, TMAZ and WZ are 92.5%, 95.0% and 88.0%, respectively.

**Figure 3:**
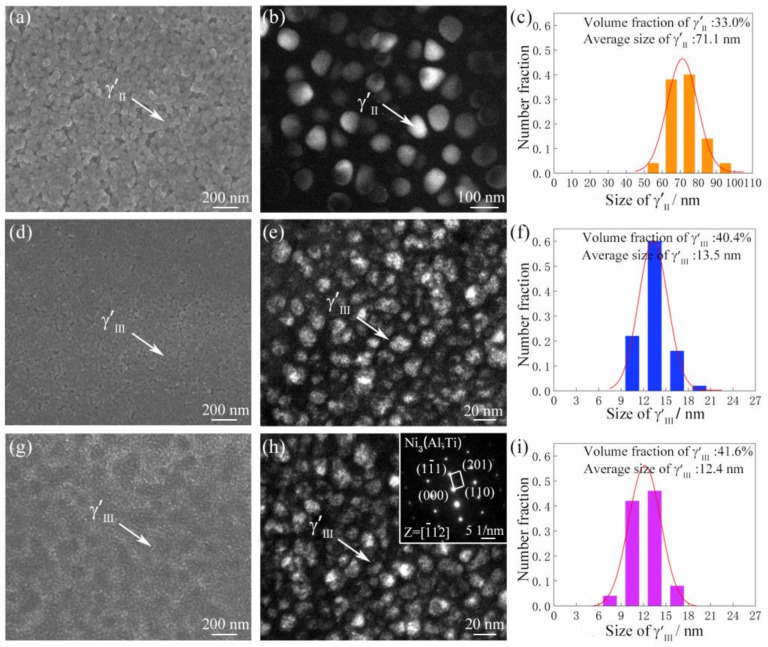
SEM results (**a**,**d**,**g**), TEM results (**b**,**e**,**h**), and size distributions (**c**,**f**,**i**) of ${\gamma^{\prime }}_{Ⅱ}$ in BM (**a**–**c**), ${\gamma^{\prime }}_{Ⅲ}$ in TMAZ (**d**–**f**) and WZ (**g**–**i**) of the GH4065A as-welded joint.

Figure 3 shows the characteristics and corresponding size and volume fraction statistics of secondary $\gamma^{\prime }$ (${\gamma^{\prime }}_{Ⅱ}$) in BM, tertiary $\gamma^{\prime }$ (${\gamma^{\prime }}_{Ⅲ}$) in TMAZ and WZ of the as-welded joint. In Figure 3a, a mass of spherical ${\gamma^{\prime }}_{Ⅱ}$ with an average size of 71.1 nm and a volume fraction of 33.0% are observed in BM (Figure 3b,c). As shown in Figure 3d–f, a large quantity of tiny ${\gamma^{\prime }}_{Ⅲ}$ with an average size of 13.5 nm and a volume fraction of 40.4% are present in TMAZ. In Figure 3g, only ${\gamma^{\prime }}_{Ⅲ}$ is observed in WZ, and its average size and volume fraction are 12.4 nm and 41.6%, respectively (Figure 3h,i). As mentioned above [12,21], the peak temperature in WZ and TMAZ is well above than the $\gamma^{\prime }$ dissolution temperature, thus the original ${\gamma^{\prime }}_{Ⅱ}$ completely dissolves into the γ matrix during welding process and fine ${\gamma^{\prime }}_{Ⅲ}$ re-precipitates in WZ and TMAZ in the following cooling process. Moreover, due to faster cooling rate and the larger temperature gradient in WZ, the ${\gamma^{\prime }}_{Ⅲ}$ re-precipitated in WZ is finer than that in TMAZ [19,22].

### 3.2. Microstructure of the PWHTed Joint

**Figure 4:**
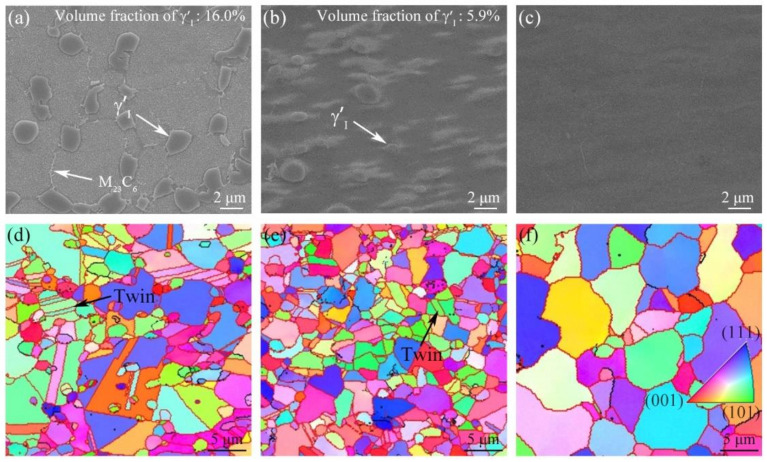
(**a**) Characteristics of ${\gamma^{\prime }}_{Ⅰ}$ (**a**–**c**) and grain structures (**d**–**f**) in BM (**a**,**d**), TMAZ (**b**,**e**), and WZ (**c**,**f**) of the GH4065A PWHTed joints after aging at 730 °C, 8 h.

Figure 4a–c show the characteristics of ${\gamma^{\prime }}_{Ⅰ}$ in BM, TMAZ and WZ of the PWHTed joint after aging at 730 °C, 8 h. The ${\gamma^{\prime }}_{Ⅰ}$ in BM of the PWHTed joint is still mainly distributed along γ grain boundaries (Figure 4a). The equivalent diameter and volume fraction of ${\gamma^{\prime }}_{Ⅰ}$ are 1–3 μm and 16.0%, respectively. In Figure 4b, the volume fraction of ${\gamma^{\prime }}_{Ⅰ}$ in TMAZ of the PWHTed joint reduces to 5.9%. Moreover, ${\gamma^{\prime }}_{Ⅰ}$ does not re-precipitate in WZ of the joint during the aging process (Figure 4c). It is obvious that the ${\gamma^{\prime }}_{Ⅰ}$ characteristics in three regions of the PWHTed joint (730 °C, 8 h) do not change significantly compared to that of the as-welded joint (Figure 2c–e). When the aging temperature is increased to 760 °C, with aging treatment for 5 h and 8 h, the characteristics of ${\gamma^{\prime }}_{Ⅰ}$ in BM, TMAZ and WZ of the PWHTed joint are still almost unchanged (see Table 2).

The characteristics of grain structures in three regions of the PWHTed joint after aging at 730 °C for 8 h are shown in Figure 4d–f. The BM and TMAZ of the PWHTed joint are still composed of equiaxed crystals and twins, and the mean grain size of BM and TMAZ are 3.42 and 2.37 μm, respectively (Figure 4d,e). The WZ of the joint is composed of equiaxed grains, and the average grain size is 6.03 μm (see Figure 4f). Besides, the proportions of HAGBs in BM, TMAZ and WZ remain relatively stable at 90.0%, 90.3% and 92.0%, respectively. The results indicate the BM, TMAZ, and WZ grain structures of the PWHTed joint (730 °C, 8 h) do not change evidently compared to that of the as-welded joint (Figure 2f–h). As the aging temperature increases from 730 °C to 760 °C, as shown in Table 2, the grain characteristics in various regions of the PWHTed joint (760 °C, 5 h) remain basically unchanged. When the holding time is prolonged to 8 h at 760 °C, the grain characteristics in various regions of the PWHTed joint (760 °C, 8 h) still do not appear observable changes. The results indicate that the IFW joint presents good grain stability during the aging treatments process.

**Figure 5:**
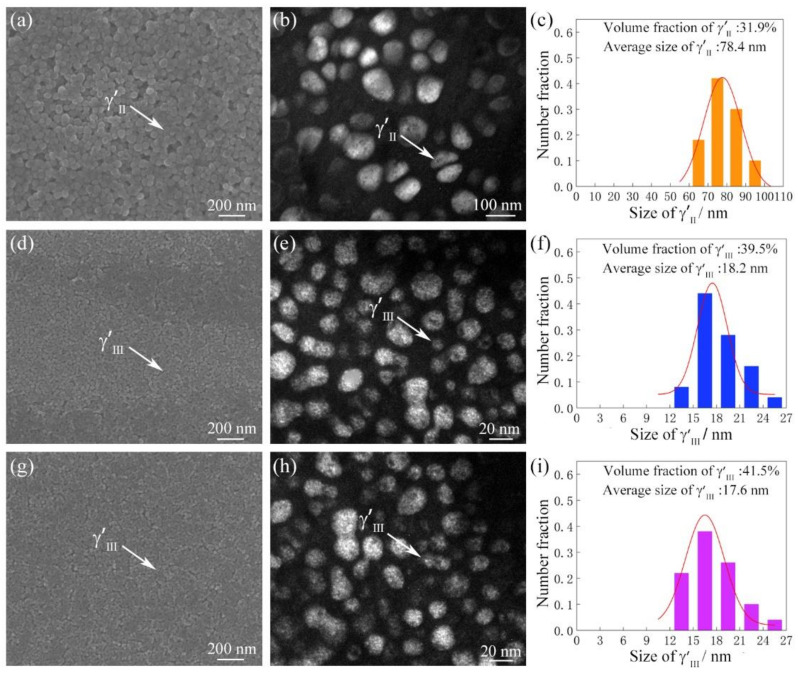
SEM results (**a**,**d**,**g**), TEM results (**b**,**e**,**h**), and size distributions (**c**,**f**,**i**) of ${\gamma^{\prime }}_{Ⅱ}$ in BM (**a**–**c**), ${\gamma^{\prime }}_{Ⅲ}$ in TMAZ (**d**–**f**) and WZ (**g**–**i**) of the GH4065A PWHTed joints after aging at 760 °C, 5 h.

Figure 5 shows the characteristics and corresponding statistics of ${\gamma^{\prime }}_{Ⅱ}$ in BM, ${\gamma^{\prime }}_{Ⅲ}$ in TMAZ and WZ of the PWHTed joints after aging at 760 °C, 5 h. The volume fraction and average size of ${\gamma^{\prime }}_{Ⅱ}$ in BM, ${\gamma^{\prime }}_{Ⅲ}$ in TMAZ and WZ of the IFW joints with different conditions are listed in Table 3. For the PWHTed joint aged at 730 °C, 8 h, the volume fraction of ${\gamma^{\prime }}_{Ⅱ}$ in BM is 32.7% and the ${\gamma^{\prime }}_{Ⅲ}$ volume fraction of TMAZ and WZ are 40.7% and 41.2%, respectively. When the aging temperature is increased to 760 °C, with aging treatment for 5 and 8 h, the ${\gamma^{\prime }}_{Ⅱ}$ volume fraction of BM remains stable at 31–33%, and the ${\gamma^{\prime }}_{Ⅲ}$ volume fraction of TMAZ and WZ reaches 39–41%. No obvious differences in the volume fraction and morphology of ${\gamma^{\prime }}_{Ⅱ}$ and ${\gamma^{\prime }}_{Ⅲ}$ are observed between the PWHTed joint and the as-welded joint (Figure 3 and Figure 5).

As seen in Table 3, for the 730 °C, 8 h PWHTed joint, the average size of ${\gamma^{\prime }}_{Ⅱ}$ in BM increases from 71.1 nm to 73.6 nm. The average size of ${\gamma^{\prime }}_{Ⅲ}$ increases from 13.5 nm to 17.4 nm in TMAZ, and from 12.4 nm to 16.7 nm in WZ. As the aging temperature increases to 760 °C, the growth of ${\gamma^{\prime }}_{Ⅱ}$ in BM and ${\gamma^{\prime }}_{Ⅲ}$ in WZ is more obvious: after aging at 760 °C, 5 h, the ${\gamma^{\prime }}_{Ⅱ}$ in BM and ${\gamma^{\prime }}_{Ⅲ}$ in WZ of the PWHTed joint grow to 78.4 nm and 17.6 nm, respectively. After aging at 760 °C, 8 h, the ${\gamma^{\prime }}_{Ⅱ}$ in BM and ${\gamma^{\prime }}_{Ⅲ}$ in WZ of the PWHTed joint grow to 85.3 nm and 19.2 nm, respectively. These results indicate that aging treatment can significantly increase the size of ${\gamma^{\prime }}_{Ⅱ}$ and ${\gamma^{\prime }}_{Ⅲ}$.

### 3.3. Creep Resistance of the As-Welded and PWHTed Joint

**Figure 6:**
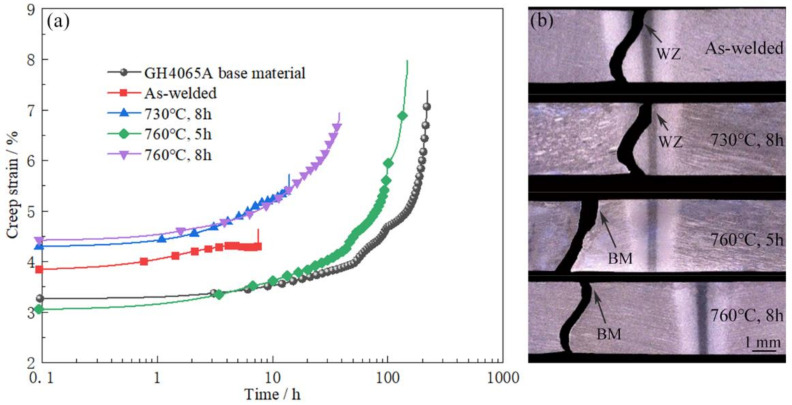
The typical creep curves (**a**) and cross sections of the crept specimens (**b**) of GH4065A base material, the as-welded joint and three PWHTed joints at 650 °C and 950 MPa.

The typical creep curves and fracture locations of the crept specimens of GH4065A base material, the as-welded joint and three PWHTed joints at 650 °C, 950 MPa are shown in Figure 6. The creep rupture time of the as-weld joint is 7.51 h, which is significantly shorter than that of GH4065A base material (221.10 h). The fracture location of the as-welded joint crept specimen locates at WZ, indicating that WZ is the weakest region of the as-welded joint at 650 °C and 950 MPa creep condition. According to the microstructural changes of BM and WZ of the as-welded joint (Figure 2 and Figure 3), it is understood that the complete dissolution of $\gamma^{\prime }$ and the small size of re-precipitated ${\gamma^{\prime }}_{Ⅲ}$ are the main reasons for the weak creep resistance of WZ. The creep rupture time of the PWHTed joint (730 °C, 8 h) extends to 13.98 h and the fracture position still locates at WZ. When the as-welded joint is aged at 760 °C, 5 h, the joint creep rupture time extends to 147.28 h and the fracture location of the joint transforms from WZ to BM, showing the creep resistance of the PWHTed joint (760 °C, 5 h) is improved significantly. According to the WZ microstructural changes of the PWHTed joints (Table 3), it is found that the growth of ${\gamma^{\prime }}_{Ⅲ}$ in WZ can improve the creep resistance of the IFW joint. However, for the 760 °C, 8 h PWHTed joint, the creep rupture time is 38.05 h, the fracture location locates at BM, and the creep resistance declines.

The other two sets of data in Table 4 show the same regularity. The creep tests results (Figure 6 and Table 4) indicate that the influence of aging at 730 °C on improving the creep resistance of joints is relatively small. Increasing the aging temperature to 760 °C promotes the strengthening effect of ${\gamma^{\prime }}_{Ⅲ}$ in WZ, which improves the creep resistance of the IFW joint. However, aging treatment with a longer time (8 h) at 760 °C may cause some damage to BM properties, resulting in the premature failure in BM during the creep process.

## 4. Discussion

In this study, aging treatment was applied to optimize the characteristics of $\gamma^{\prime }$ and enhance the creep resistance of the GH4065A IFW joint. The grain structures and ${\gamma^{\prime }}_{Ⅰ}$ in BM, TMAZ and WZ of the GH4065A IFW joint exhibited very little variation during the aging treatment processes (730 °C for 8 h, 760 °C for 5 h, 760 °C for 8 h) due to the high thermal-stability of GH4065A alloy [23] (see Figure 4). Post-welding aging treatment promoted the growth of ${\gamma^{\prime }}_{Ⅲ}$ in WZ, which increased from 12.4 nm to 17.6 nm after 760 °C, 5 h aging treatment. The growth of the ${\gamma^{\prime }}_{Ⅲ}$ in WZ increased the ${\gamma^{\prime }}_{Ⅲ}$ strengthening effect and improved the creep resistance of the PWHTed joint (Table 3 and Figure 6). Meanwhile, as seen in Table 3 and Figure 6, the excessive growth of ${\gamma^{\prime }}_{Ⅱ}$ in BM was detrimental to BM properties [24], resulting in the premature creep failure at BM of the PWHTed joint (760 °C, 8 h).

The $\gamma^{\prime }$ evolution in Ni-based superalloy IFW joint was closely related to the thermal welding process. The peak temperature at the friction interface was obviously higher than the $\gamma^{\prime }$ dissolution temperature [19,20], resulting in the complete dissolution of $\gamma^{\prime }$ in WZ during the IFW process. For high-alloyed Ni-based superalloys, the WZ quickly reached the supersaturation state for $\gamma^{\prime }$ forming elements during the welding cooling process. Meanwhile, high supercooling offered a great impetus for the precipitation of $\gamma^{\prime }$, resulting in the re-precipitated ${\gamma^{\prime }}_{Ⅲ}$ in WZ being extremely fine and having a high-volume fraction [7]. During the aging treatment process, the ${\gamma^{\prime }}_{Ⅱ}$ in BM, ${\gamma^{\prime }}_{Ⅲ}$ in TMAZ and WZ would grow to different sizes, mainly determined by the diffusion rate of the alloying elements and generally following the Lifshitz–Slyozov–Wagner (LSW) relationship [25,26]. Additionally, aggregation growth was also an essential growth mechanism for ${\gamma^{\prime }}_{Ⅲ}$ in WZ of the IFW joint during aging treatment [27].

The ${\gamma^{\prime }}_{Ⅲ}$ volume fraction in WZ was a crucial factor for enhancing the properties of Ni-based superalloy IFW joint [4,5]. Increasing the volume fraction of $\gamma^{\prime }$ decreased its spacing in the γ matrix, resulting in increased resistance to dislocation movement [28]. As can be observed from Table 3, the post-welding aging treatment had no significant impact on the volume fraction of ${\gamma^{\prime }}_{Ⅲ}$ in WZ. In detail, the ${\gamma^{\prime }}_{Ⅲ}$ volume fraction in WZ of IFW joint was ~41%, and the volume fraction kept relatively stable at 41–42% after the aging treatment. The ${\gamma^{\prime }}_{Ⅲ}$ volume fraction in WZ depended on the welding cooling process, and the ${\gamma^{\prime }}_{Ⅲ}$ volume fraction exhibited an increasing trend as the cooling rate decreased [29]. The subsequent aging treatment could only supplement a small amount of ${\gamma^{\prime }}_{Ⅲ}$ in WZ of the PWHTed joint, which did not have a significant impact on the volume fraction of ${\gamma^{\prime }}_{Ⅲ}$ in WZ [7]. It is noteworthy that during the IFW process, severe plastic deformation of WZ material promoted the nucleation and massive precipitation of ${\gamma^{\prime }}_{Ⅲ}$, making it exhibit the characteristics of explosive nucleation [12]. Therefore, for the Ni-based superalloys with high content of $\gamma^{\prime }$, it was difficult to nucleate and re-precipitate ${\gamma^{\prime }}_{Ⅲ}$ in WZ during the post-welding aging treatment [21].

During the post-welding aging treatment, the growth of ${\gamma^{\prime }}_{Ⅲ}$ in WZ can effectively improve the high-temperature properties of the Ni-based superalloy IFW joint. For example, the tensile yield strength of the FGH96 IFW joint increased by approximately 80 MPa because the ${\gamma^{\prime }}_{Ⅲ}$ in WZ grew from 16 nm to 20 nm after aging treatment [7]. For U720Li Ni-based superalloy with 35% of $\gamma^{\prime }$, the critical resolved shear stress (CRSS) of the alloy increased with the $\gamma^{\prime }$ size when the size was less than 40 nm. However, when the size of $\gamma^{\prime }$ was above 40 nm, the CRSS decreased with its size [30]. Moreover, the size of $\gamma^{\prime }$ affected the alloy deformation mechanism. When $\gamma^{\prime }$ was small, the deformation mechanism was the shearing mechanism (dislocation shearing $\gamma^{\prime }$), and the $\gamma^{\prime }$ strengthening effect increased with the $\gamma^{\prime }$ size. However, when the $\gamma^{\prime }$ grew to a certain range, the deformation mechanism transformed into Orowan looping (dislocation bypassing $\gamma^{\prime }$), and the $\gamma^{\prime }$ strengthening effect decreased with its size [24].

**Figure 7:**
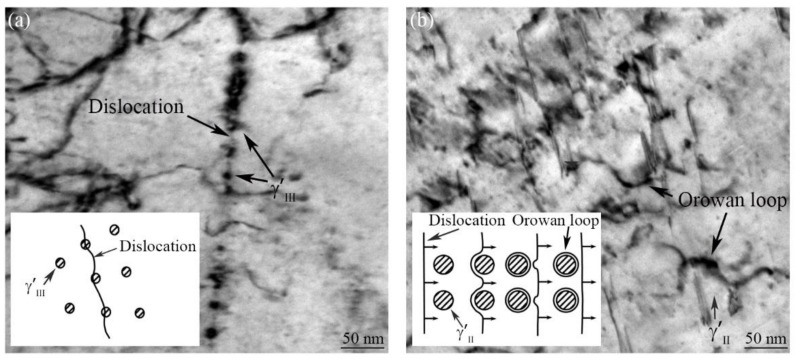
TEM results of the creep fracture location of the as-welded joint (**a**) and the PWHTed joint (760 °C, 8 h) (**b**).

The interaction between $\gamma^{\prime }$ and dislocation in the creep fracture region of the GH4065A as-welded joint is shown in Figure 7a; it is seen that the dislocations shear ${\gamma^{\prime }}_{Ⅲ}$ in WZ during the creep deformation process. For the shearing mechanism, the strengthening effect of ${\gamma^{\prime }}_{Ⅲ}$ mainly depends on the CRSS of the dislocations shearing ${\gamma^{\prime }}_{Ⅲ}$. The CRSS is calculated as follows [31]:

(1) $$\tau_{s}={\left(r_{0}/b\right)}^{3/2}{\left(4fr_{s}/\left(\pi T\right)\right)}^{1/2}$$

where $\tau_{s}$ is the CRSS of dislocations shearing ${\gamma^{\prime }}_{Ⅲ}$, *r_0_* is the antiphase boundary (APB) energy per unit area, *b* is Burgers vector, *f* is the volume fraction of ${\gamma^{\prime }}_{Ⅲ}$, *r_s_* is the average size of the ${\gamma^{\prime }}_{Ⅲ}$, and *T* is the dislocation line tension. The results of Figure 3 and Figure 5 indicated that when the ${\gamma^{\prime }}_{Ⅲ}$ volume fraction in WZ and other conditions were basically the same, the increase of ${\gamma^{\prime }}_{Ⅲ}$ size within a certain range increased the CRSS of dislocations shearing ${\gamma^{\prime }}_{Ⅲ}$, which in turn increased the ${\gamma^{\prime }}_{Ⅲ}$ strengthening effect.

The TEM image of the creep fracture location of the PWHTed joint (760 °C, 8 h) is shown in Figure 7b. The dislocations bypass the ${\gamma^{\prime }}_{Ⅱ}$ and form Orowan loops in BM during the creep deformation process. For Orowan looping, the ${\gamma^{\prime }}_{Ⅱ}$ strengthening effect is mainly influenced by the CRSS of dislocations bowing. The CRSS can be described as follows [32]:

(2) $$\tau_{o}=Gb/L$$

where $\tau_{o}$ is the CRSS of dislocations bowing, *G* is the shear modulus, and *L* is the spacing between ${\gamma^{\prime }}_{Ⅱ}$ [32]:

(3) $$L=r_{s}\left(\sqrt{8/3\pi f}-1\right)$$

where *r*_s_ is the average size of ${\gamma^{\prime }}_{Ⅱ}$. According to the model (2) and (3), increasing the ${\gamma^{\prime }}_{Ⅱ}$ size would decrease the CRSS of dislocations bowing. Therefore, the growth of ${\gamma^{\prime }}_{Ⅱ}$ in BM of the PWHTed joint (760 °C, 8 h) weakened the strengthening effect of ${\gamma^{\prime }}_{Ⅱ}$, thus decreasing the creep resistance of BM (Table 3 and Figure 6).

**Figure 8:**
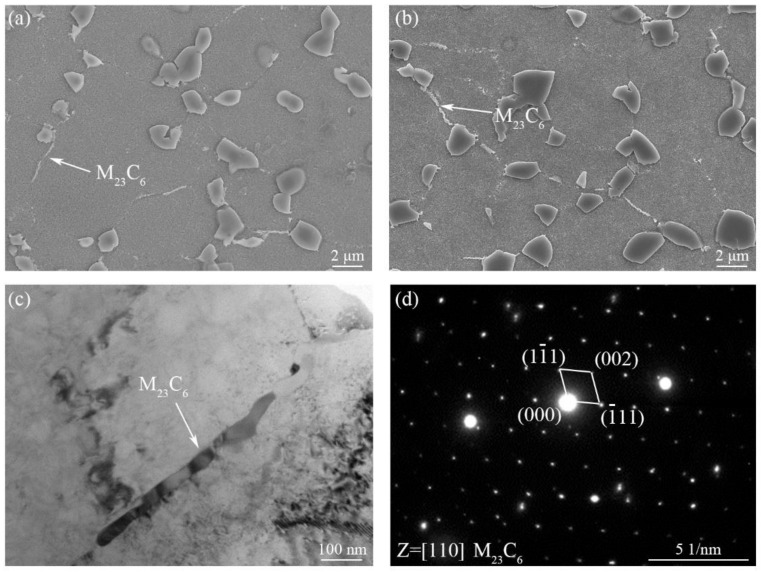
SEM images in BM of the GH4065A PWHTed joints after aging at 760 °C, 5 h (**a**) and 8 h (**b**), TEM result (**c**) and SAED pattern (**d**) of the new precipitate of BM.

Besides, M_23_C_6_ carbides precipitated at grain boundaries had a significant impact on the properties of Ni-based superalloys. The characteristics of M_23_C_6_ carbides in BM of the IFW joints after aging at 760 °C for 5 h and 8 h are shown in Figure 8. New precipitates are observed at the grain boundaries in BM of the PWHTed joint, which show a tendency to increase and grow as aging temperature or time increases (see Figure 4a and Figure 8a,b). The TEM and selected area electron diffraction (SAED) pattern results show that the precipitates are M_23_C_6_ (Figure 8c,d). M_23_C_6_ particles were common carbides in Ni-based superalloys and generally precipitated at the grain boundaries during aging treatment [33,34]. The precipitation and growth of M_23_C_6_ carbides at the grain boundaries were promoted by increasing aging temperature or prolonging aging time [1,33]. The discrete M_23_C_6_ carbides improved the high-temperature strength of alloys by hindering grain boundaries sliding. However, the continuous M_23_C_6_ carbides weakened the bonding force between grains, thus reducing the high-temperature properties of the material [34]. Therefore, the continuous M_23_C_6_ carbides in BM of the PWHTed joints may be the reason for the decrease of creep properties of BM of the joint.

## 5. Conclusions

In this work, the effect of aging treatment on the microstructure and creep resistance of the GH4065A IFW joint was systematically investigated. The main conclusions are as follows:

1. After aging treatment, the size of ${\gamma^{\prime }}_{Ⅲ}$ in WZ and ${\gamma^{\prime }}_{Ⅱ}$ in BM of the IFW joint increased, but their morphology and volume fraction did not change evidently. Meanwhile, aging treatment did not significantly change the characteristics of grain and ${\gamma^{\prime }}_{Ⅰ}$ in BM, TMAZ and WZ of the IFW joint;
2. The 730 °C, 8 h aging treatment had little effect on improving the creep resistance of the GH4065A IFW joint. The 760 °C, 5 h aging treatment significantly enhanced the creep resistance of the IFW joint, and the creep rupture time of the joint at 650 °C and 950 MPa increased from 7.51 h to 147.28 h. After extending the aging time to 8 h at 760 °C, the creep rupture time of the joint was reduced to 38.05 h;
3. After aging at 760 °C, 5 h, the ${\gamma^{\prime }}_{Ⅲ}$ in WZ of the IFW joint grew from 12.4 nm to 17.6 nm. The growth of ${\gamma^{\prime }}_{Ⅲ}$ in WZ of the joint during the aging treatment process was the primary reason for improving the creep resistance of the IFW joint. However, with a higher temperature and a longer aging time, the further growth of ${\gamma^{\prime }}_{Ⅱ}$ in BM and the continuous precipitation of M_23_C_6_ carbides at the grain boundaries of BM might reduce the creep resistance of BM of the joint.

## Figures and Tables

**Table 1:** Chemical composition of GH4065A (wt.%).

C	Cr	Co	Al	Ti	Nb	Mo	W	Ni
0.015	16.0	13.0	2.1	3.7	0.7	4.0	4.0	Bal.

**Table 2:** Volume fraction of ${\gamma^{\prime }}_{Ⅰ}$, average grain size and proportions of HAGBs in three regions of the IFW joints with different conditions.

PWHT	Region	$\mathrm{Volume}\;\mathrm{Fraction}\;\mathrm{of}\;{\gamma^{\prime }}_{Ⅰ}(\%)$	Average Grain Size (μm)	Proportion of HAGBs (%)
As-welded	BM	15.1 ± 1.7	3.66 ± 0.23	92.5 ± 3.6
	TMAZ	5.5 ± 1.3	2.86 ± 0.40	95.0 ± 1.1
	WZ	/	4.88 ± 0.49	88.0 ± 4.3
730 °C, 8 h	BM	16.0 ± 1.1	3.42 ± 0.30	90.0 ± 4.7
	TMAZ	5.9 ± 0.9	2.37 ± 0.35	90.3 ± 4.3
	WZ	/	6.03 ± 0.63	92.0 ± 3.2
760 °C, 5 h	BM	15.0 ± 2.0	3.12 ± 0.46	94.0 ± 3.5
	TMAZ	5.2 ± 1.5	2.72 ± 0.21	94.6 ± 2.9
	WZ	/	5.68 ± 0.58	95.1 ± 2.3
760 °C, 8 h	BM	15.1 ± 1.6	3.53 ± 0.19	86.5 ± 4.1
	TMAZ	5.7 ± 1.1	2.70 ± 0.26	94.5 ± 2.5
	WZ	/	5.10 ± 0.38	93.4 ± 3.8

**Table 3:** Volume fraction and average size of ${\gamma^{\prime }}_{Ⅱ}$ in BM, ${\gamma^{\prime }}_{Ⅲ}$ in TMAZ and WZ of the IFW joints with different conditions.

PWHT	Region	$\mathrm{Volume}\;\mathrm{Fraction}\;\mathrm{of}\;{\gamma^{\prime }}_{Ⅱ}(\%)$	$\mathrm{Volume}\;\mathrm{Fraction}\;\mathrm{of}\;{\gamma^{\prime }}_{Ⅲ}(\%)$	$\mathrm{Average}\;{\gamma^{\prime }}_{Ⅱ}\mathrm{Size}\;(\mathrm{nm})$	$\mathrm{Average}\;{\gamma^{\prime }}_{Ⅲ}\mathrm{Size}\;(\mathrm{nm})$
As-welded	BM	33.0 ± 1.1	/	71.1 ± 1.5	/
	TMAZ	/	40.4 ± 1.6	/	13.5 ± 0.4
	WZ	/	41.6 ± 1.4	/	12.4 ± 0.5
730 °C, 8 h	BM	32.7 ± 0.7	/	73.6 ± 1.2	/
	TMAZ	/	40.7 ± 0.8	/	17.4 ± 0.7
	WZ	/	41.2 ± 1.7	/	16.7 ± 0.5
760 °C, 5 h	BM	31.9 ± 1.4	/	78.4 ± 1.9	/
	TMAZ	/	39.5 ± 1.9	/	18.2 ± 1.0
	WZ	/	41.5 ± 0.7	/	17.6 ± 0.9
760 °C, 8 h	BM	32.5 ± 1.0	/	85.3 ± 2.3	/
	TMAZ	/	40.2 ± 1.3	/	20.8 ± 1.3
	WZ	/	41.8 ± 1.1	/	19.2 ± 1.1

**Table 4:** The creep properties of GH4065A base material, the as-welded joint and three PWHTed joints at 650 °C and 950 MPa.

Condition	Number	Creep Rupture Time (h)	Strain (%)	Fracture Location
GH4065A base material	1	221.10	7.38	/
	2	234.41	7.51	
	3	208.33	7.09	
As-welded	1	7.51	4.63	WZ
	2	6.59	4.37	
	3	5.64	4.03	
730 °C, 8 h	1	13.98	5.72	WZ
	2	10.36	5.29	
	3	12.97	5.54	
760 °C, 5 h	1	147.28	7.98	BM
	2	154.31	8.11	
	3	138.16	7.77	
760 °C, 8 h	1	38.05	6.94	BM
	2	46.23	7.54	
	3	32.53	6.79	

## Data Availability

Not applicable.
